# Student-faculty interactions within a physiotherapy curriculum in South Africa

**DOI:** 10.1007/s10459-021-10070-x

**Published:** 2021-09-23

**Authors:** Serela S. Ramklass, Renuka Vithal

**Affiliations:** 1 School of Clinical Medicine, College of Health Sciences, University of Kwazulu-Natal, 719 Umbilo Road, Durban, 4013 South Africa; 2 Faculty of Education, University of Fort Hare, Private Bag X1314, King Williams Town Road, Alice, 5700 South Africa

**Keywords:** Caring, Physiotherapy curriculum, Student-faculty interactions

## Abstract

Interactions between faculty and students in higher education has the potential to influence and shape many aspects of teaching, learning, curricula, student experiences and performance, yet has received little attention as an area of study. This study investigates student-faculty interactions within a physiotherapy curriculum from the perspectives of students, faculty and physiotherapy managers at a South African university. The data, produced through multiple methods, derive from students, faculty and physiotherapy managers underpinned by critical-feminist perspectives. Thematic analysis of the data produced four themes. Two dominant threads emerging from the analysis as characterising student-faculty relationships are the deeply hierarchical relations of power characterised by a lack of caring and concern for students, and the exclusion of wider constructs for interaction; deriving from a particular entrenched medical model. Ironically, while caring relationships with patients are overtly advocated and developed, they appear to be largely absent in the same physiotherapy curriculum spaces in the relationships between faculty and students. These findings raise questions about how the most foundational attribute of a health science professional, that of caring, is being produced through the curriculum in the relationship between faculty and students in the health sciences.

## Introduction and literature

Teaching and learning is a relational process that is influenced by the interactions between student and faculty, these relationships affecting student satisfaction and the effectiveness of learning. Health professionals pride themselves in nurturing caring in their students as part of the higher education curriculum to ensure that once students go into practice, they will transfer the caring behaviours and attributes to the relationships they develop with their patients. It is hoped that these caring behaviours and attributes become re-inscribed in settings and relationships outside the university learning environment, for example, when the authoritative role shifts to the student in practice settings. This study, therefore, explored how caring was produced in the interactions between students and faculty in the physiotherapy classroom and in clinical practice settings.

The first objective of the literature review was to identify and synthesise international literature on the nature, purpose and critical elements related to student-faculty interactions by undertaking a focused review. The second was to provide an overview of the South African socio-political context, particular to higher education and healthcare that has shaped the development of interactions amongst students, faculty and patients. An environmental scan of reports, literature and regulatory processes was undertaken to describe the historical and current context of the practice of South African higher education and healthcare.

## Student-faculty interactions

An attribute of successful teaching is the strength of the interpersonal relationships that develop between students and faculty, the effectiveness of which can have implications for the success or failure thereof (Doncan-Morgan, 2009). Students have reported that student-faculty interaction inside and out of class affected their intellectual achievements, influenced their selection of courses and satisfaction with their college experience (Cuseo, 2018). ‘Care’ and ‘boundaries’ describe the dynamic interplay of student-faculty interactions, holding the interaction together in gravitational tension (Ross, 2013).

Hagenauer and Volet (2014) identify two main dimensions that can be differentiated when describing teacher-student relationships (TSR) in higher education. These are the *affective dimension*, which describes the bond built between students and teachers that forms the basis for secure and affective, positively experienced relationships, and the *support dimension*, which describes the support that must be provided through TSR for students’ success (e.g. teachers setting clear expectations, answering emails promptly).

Of the different aspects that comprise the affective dimension in teacher-student relationships, Hagenauer and Volet (2014) highlight one particular aspect, that of “caring for students”, as having strong empirical support in the general literature as a humanistic value. Humanistic attributes for healthcare include active listening, displaying empathy and compassion, having meaningful communication, displaying sensitivity and respect, all of which have long been valued but received little or no formal attention in health sciences curricula (Benatar, 2015; Reid, 2014).

Noddings (2012) recognises the powerful role of the educator in developing caring relationships, particularly in professions that are care-related, such as education. She describes four components for educators that are central to developing caring relationships. These are (a) *modelling* i.e. showing in the behaviour as teachers what it means to care; (b) *dialogue* is the way to model the caring ideal in communication; (c) *practise*, where the teacher models caring communication while the student practises it, and (d) *confirmation*, where the teacher identifies a better self in the student and encourages its development (Bergman, 2004). Skills for interpersonal relationships are strongly influenced by positive role models, and student-faculty relationships, which can be a model within the health sciences disciplines for care relationships with patients.

Health professional educators are increasingly challenged to produce graduates who espouse values that are responsive to the desired outcomes of the healthcare system in terms of patient care. These values have been given prominence in official and formal healthcare curricula to extend the notion of patient-care, resulting in a stronger focus on the nature of relationships between students-in-training and patients, and therefore on the graduate attributes and values that a curriculum produces. This has been identified as requiring attention in health sciences curricula to ensure competent and caring relationships between soon-to-become practitioners and their patients (Health Professions Council of South Africa, 2014).

In addition to its two-dimensional nature, Hagenauer and Volet (2014) found that TSR must be regarded as a *context-dependent* construct, which can be interpreted in a number of ways. For example, Asian TSRs and expectations may differ from Western ones due to their varying cultural practices related to contextual factors (Hagenauer and Volet, 2014). Another interpretation of context dependency relates to the disciplinary context. The disciplinary context of an academic community is defined, amongst others, by the faculty, which varies across disciplines (Ross, 2013).

## South African socio-political context

Healthcare and education in South Africa were racially segregated during the pre-democracy period of apartheid up to 1994, having been used as an instrument of privilege and control for those categorised as Whites that centred on themes of racial and cultural purity and superiority (Verwy and Quayle, 2012). Apartheid education entrenched a particular pedagogical ideology that viewed the TSRs in terms of a deeply paternalistic parent-child relationship (Kallaway, 2002).

Challenges in the current higher education context include racism and discrimination, language and institutional cultures that serve to exclude rather than empower, and high failure rates which are linked to inadequate support for poor Black students (Department of Higher Education and Training, 2016). Students at a historically Black university in South Africa, alluded to current tensions produced by South Africa’s history, race and class dynamics that limit racial interactions and shape relations of marginalisation and privilege (Bhana, 2014). These dynamics produce hostilities and resentment towards those students and faculty perceived as privileged; these being the Whites, the middle/elite classes of Indians and also those ‘superior Blacks who think they have everything’ (Bhana, 2014).

Students in the health sciences may relate differently to faculty compared to those in other disciplines due to the nature of the health sciences curriculum creating various imperatives for student-faculty relationships in different contexts. For example, in practical training and in clinical education settings where the closer relationships during small group teaching sessions are characteristic of health science curricula. However, there is a silence on the concomitant relationships between health sciences faculty and their students in the enactment of the very same curriculum.

Caring is central to the practice of physiotherapy. Ramklass (2015) details multiple framings of caring for South African physiotherapy practice and education that are important for relationship development given the country’s socio-political history. These components of caring identified for physiotherapy include collegiality and valuing; listening and showing empathy and nurturing; being an expert practitioner; having interdisciplinary knowledge and demonstrating biopsychosocial interventions; having cultural and language competence, and showing human and community connectedness.

Altruism, equality and capability have also been identified as common health practitioner values in the literature (Moyo et al., 2016). Specific altruistic values include caring, compassion and empathy; values of equality include respect, human dignity and social justice whilst values of capability include excellence, competency and knowledge (Moyo et al., 2016).

This paper casts a research gaze on one health science discipline, that of physiotherapy in one higher education institution in South Africa, with a specific focus on the interactions between faculty and students. The relationships between students and faculty within a physiotherapy curriculum are examined from multiple vantage perspectives of the students, faculty themselves and physiotherapy managers at one South African university. This study and the framings of caring, both derive from a larger study conducted by the first author while employed as physiotherapy faculty. The study examined how a South African physiotherapy curriculum had responded to the country’s socio-political transformation post-apartheid, and questioned further, why it responded in the way that it did. The data, produced through multiple methods, are from students, faculty and physiotherapy managers underpinned by critical-feminist perspectives. This paper draws on the same data source and focuses specifically on one aspect, that of student-faculty interactions in the physiotherapy curriculum.

## Methodology

This qualitative research design was framed within narrative inquiry, underscored by critical and feminist perspectives. Narrative inquiry tends to be associated with emancipatory approaches (Gough, 1998) and has the potential to construct caring communities by sharing stories (Noddings, 1991). Critical and feminist perspectives are grounded in conceptions of power as the possession of dominant and repressive forces that are used to dominate, oppress, coerce and deny (Gore, 1993). Stone (1995) states that both theories are committed to emancipation and empowerment of persons, and the recognition of the centrality of ideologies of power and change. Both are cognisant of the importance of education as a site of liberatory struggle for consciousness-raising and transformative social action. Feminist perspectives are widely deployed in studies of caring in education (Noddings, 1989).

The study participants included physiotherapy faculty, final year physiotherapy students and physiotherapy managers based at clinical training sites. Further description of participants include details of their race and gender which are relevant to the analysis. Apartheid South Africa defined four major race groups of Black African, Whites, Indians, (derived from migrants from the Indian sub-continent), and Coloureds^1^, (persons of mixed race), which still persists. Data collection methods included semi-structured interviews (faculty), focus group interviews and journaling (final year students), and a group interview (physiotherapy managers).

Individual semi-structured interviews were held with seven of the nine faculty members, who comprised one Black African, five Indians and one Coloured, which consisted of one male and six females.

Two in-depth focus groups interviews together with journal entries were used to produce data from 42 physiotherapy final year students. Focus group interviews were scheduled, one before and one after the elective clinical block. An elective is a three-week clinical education block in the final year of the programme, during which students practise physiotherapy at a clinical site of their choice. Pre-elective focus group interviews were conducted at five clinical education sites, and are referred to in the analysis as A1, B1, C1, D1 and E1. Post-elective focus group interviews were conducted at three clinical education sites and are referred to as A2, D2 and E2. The distribution of participants into smaller groups is indicated in the analysis as focus group 1 (FG1) or focus group 2 (FG2).

The cohort comprised 36 female students: 17 Indian, 11 Black, six White and two Coloured. Of the six male students, two were Indian and four were African. Each student was given a journal to document his/her experiences of becoming a health care professional within their undergraduate program, of which 29 were returned, with guided reflection being used to assist the journal entries.

A group interview was conducted with 19 physiotherapy managers from public hospitals. There were 2 Black males, while the 17 females comprised eleven Indian, one Coloured, two Black and three White. Twelve of the participants were experienced physiotherapists who occupied management positions at urban, well–resourced public hospitals or health care centres, having had over five years of working experience. Seven participants were novice physiotherapists at small, rural, under-resourced hospitals.

Data collection focused on how caring in interpersonal relationships were constructed and experienced between student physiotherapists and faculty, and observed by physiotherapy managers. Multiple data collection methods, data sources and member checks were used to assure validity, with the interviews and focus group discussions being audio-taped and transcribed. Data triangulation through consensus amongst the multiple data sources, as well as transparency and honesty relative to the context, were important to assure trustworthiness (Demarrais & Lapan, 2004).

Thematic analysis of the transcripts and journals was undertaken. The process involved coding and theme generation. Data from each participant was first individually coded and grouped into common patterns. The themes generated from each data source were reviewed, first individually and then collectively, to identify similarities and divergences.

The article therefore sheds light on and explores how caring was produced in the interactions between students and faculty in physiotherapy, as reported by them and observed by physiotherapy managers.

## Findings

The findings with evidence from the data are presented from three perspectives of: the students; the faculty, and the observations of physiotherapy managers from the practice settings. Using a deductive analytic approach, the themes draw on Ramklass (2015) multiple framings of caring for South African physiotherapy practice and education. The focus in the analysis is on the different aspects and features that emerged in characterising the relationship between students and faculty. In this explication are also the reasons advanced or solutions proposed for addressing concerns and critique thereby improving the relationships. Themes that emerged from the analysis are (1) displaying caring by listening, showing empathy and nurturing, (2)interpersonal relationships, collegiality and interconnectedness, (3) cultural and language competence, and (4) enacting interdisciplinary knowledge and demonstrating biopsychosocial interventions.

1. A theme that was central to the data was that of displaying caring by listening, showing empathy and nurturing. Physiotherapy was perceived as “a caring profession” (Faculty D) and “caring should be the guiding principle for training physiotherapists” (Faculty B). The “identity of the profession would be lost if caring was absent” (Faculty B).
  While a few faculty espoused how caring was constructed in their “approach to teaching as a parent” (Faculty D) and as “the nurturing mother” (Faculty G), others reported that despite physiotherapy being perceived as a caring profession, they did not demonstrate caring. They “do not give students love and care, to open and blossom” (Faculty G), “students think that we’re so far away from them, that we can’t reach them”* (Faculty F), and *“there are students who think that there are lecturers who do not care for them” (Faculty D). The absence of caring by faculty was confirmed by student reflection in the quote “it seems like no-one cares for us” (Student focus group interview: A1FG2).
  Listening, showing empathy and nurturing, were generally not experienced by students, while several faculty conceded that these were lacking in their relationships with students. Students reported that “lecturers don’t stop to listen to you and you have a whole lot of issues.” (Student focus group interview: D1FG2). Faculty agreed with this view saying, “there is no time to get the student’s perspective. We just try to push the syllabus.” (Faculty D). Students stated that faculty were not empathetic towards their needs and that “lecturers need to improve their approach towards students that are struggling” (Student focus group interview: A1FG1).
  Faculty agreed that to develop caring in students as part of the professional identity, it had to be modelled during teaching in the classroom and clinical environment, and in their interactions with students. Modelling care was imparted by the educator in “role-playing on how to handle, assess and treat a patient” (Faculty B). Students, on the other hand, described their difficulty in modelling faculty because “You hardly see them [faculty] doing hands-on or showing you how to do this or that. You have nothing to fall back on or visualise or try out because you have never seen them do it” (Student focus group interview: D1FG1).
2. Developing interpersonal relationships, collegiality and interconnectedness between students and faculty, faculty and physiotherapy managers and amongst faculty themselves featured as another dominant theme. Student physiotherapists were clear about the importance of skills for interpersonal relations as part of their professional identity, “If you can’t have an interpersonal relationship, you can’t be a physio” (Student focus group interview: E1FG1). However, these skills were not considered important and there were no explicit opportunities designed within the curriculum to develop interpersonal relationships. Communication amongst students, and between students and faculty was poor, “there is a divide between students and lecturers” (Student focus group interview: D2FG1).
  Students stated that “nobody takes our viewpoint into consideration” (Student focus group interview: D1FG1), while faculty described how their relationships lacked collegiality and valuing. This was evident as they did not make an effort “to meet and mix” (Faculty D), “to discuss how we teach” (Faculty E), “to share ideas” (Faculty A), and they reflected on how these behaviours were “rubbing off onto the students” (Faculty A). The state of the student-faculty relationship rested on the relationships amongst faculty themselves, the absence of cohesion and operating in isolation from each other, which was being transferred to students.
  Student-faculty interactions were characterised by hierarchical authoritarianism, disrespect, silencing, and ‘othering’ arising from discrimination. The students described faculty as “intimidating” (Diary No. 18) and disrespectful, particularly during clinical examination sessions where faculty “make students feel small and degrade them” (Student focus group interview: C1FG2). Authoritarianism featured in student-faculty interactions, which functioned to silence students. “Being in the physiotherapy department feels like being in school…the lecturers have some authority, some hold over you. I used to be so enthusiastic in the first year, raising my hands to answer questions, but now it’s about maintaining a low profile, obtaining my degree and leaving” (Student focus group interview: C1FG2). Authoritarianism was also observed by the physiotherapy managers, “lecturers must come down to the level of the students and they need to be open to suggestions. Most of these students are so afraid to ask questions” (Participant: Group A), and by faculty themselves, “The moment they see us, a figure of authority, they freeze.” (Faculty G).
  A strong theme from the perspective of faculty, that underscored student-faculty interactions, was pedagogical authority described primarily as a parent-child association. Several faculty referred to this conception in how they related to students: “To me, they are not really students. They are children. When they are at university, I stand in for their parents” (Faculty D). “I always look at them and say, ‘you’re children.’ Those that I show caring for, I can discipline very nicely because they know I care” (Faculty F).
  Faculty authority extended to acts of discrimination along the lines of race. Students described how “certain students are being favoured…more African students are experiencing problems than maybe Indians, Coloureds, or Whites” (Student focus group interview: C1FG2), and that there was a “lack of consistency in how people are treated” (Student focus group interview: D1FG1). Amongst students, there was “racial tension between the races and amongst the races themselves in all levels of study” (Student focus group interview: D1FG1). Faculty was aware of the lack of racial integration amongst students and consciously acted on breaking barriers during group work sessions by “…dictat[ing] the groups and make sure that they are racially interspersed so there is no discrimination of race or gender. So we learn to integrate amongst each other, learn to appreciate and respect the next person” (Faculty F).
  Relations of power underscored the element of caring related to collegiality and interconnectedness, which was recognised by all participants but lacking in action. Caring as human interconnectedness requires an examination of assumptions, biases and subjectivities concerning race, class, gender, culture, language and power. The students indicated that they lacked a feeling of interconnectedness and felt that they did not belong to the department (Student focus group interview: D1FG1).
  Students reported that “the department should do something to unite students and lecturers and build good relationships, and improve contact between students and lecturers, and between the students themselves” (Student focus group interview: D1FG1). These could be achieved by, for example, forming sports teams within the department (Diary No. 12), initiating bonding/teamwork sessions from the first year (Diary No. 8), or through talks on improving relationship dynamics (Student focus group interview: C1FG1) and the initiation of a buddy system (Student focus group interview: C1FG1).
  Faculty perceived that the environment contributed negatively to developing interpersonal skills between themselves and students due to the absence of a common meeting place, “If we had a common room, it would make so much difference, because we could walk in when there are students and we could have a few laughs with them…even amongst students themselves there isn’t that cohesion” (Faculty D). Physiotherapy managers also alluded to poor interaction of faculty with hospital staff and how this influenced the relevance of caring in the curriculum. Faculty were perceived as being “out-dated in relation to what’s happening in the clinical environment and they should come out more, do more hands-on and interact with the consultants” (Participant: Group C).
  The dominant views that emerged about interpersonal relationships in physiotherapy are characterised as being divided, with a lack of communication and interaction. Students perceived the importance of developing skills for interpersonal relationships within the curriculum for translation into patient-therapist interactions. It is evident that while students are critical, embedded in their critique are suggestions for solutions, such as more communication and engagement in extra-curricular social events to enhance the relations between themselves and faculty.
3. The third theme, cultural and language competence, has a potential role in advancing interpersonal relations. Both students and physiotherapy managers showed awareness that language and cultural differences presented barriers to developing interpersonal relationships. Students observed how their “inability to speak and understand isiZulu was limiting during interactions with patients at the hospital” (Student focus group interview: A1FG1) yet “the physiotherapy programme offered nothing to improve one’s skills when interacting with a patient” (Student focus group interview: B1FG2). It was suggested that “It would help if we could have lectures on how to incorporate the different cultures; different races… because when there is contact between a white and black person, there are always differences” (Student focus group interview: E1FG1). Physiotherapy managers concurred with this finding stating that “Students have to know about the different Black cultures to maximise their treatment. Students must be made aware that they must learn isiZulu, especially if you’re not an isiZulu speaking person.” (Participant: Group E).
4. The fourth theme, enacting interdisciplinary knowledge and demonstrating biopsychosocial interventions, was considered important by all participants for the patient-practitioner relationship. However, the interaction between physiotherapy students and students from other health sciences disciplines did not feature in the curriculum, this being foundational for an interdisciplinary, holistic practice. Students indicated that “there was no interaction with students from within the School” (Student focus group interview: A1FG2). Faculty resonated with this finding, indicating “we talk of interdisciplinary, holistic treatment, but we’re not even doing it in our teaching” (Faculty G). Physiotherapy managers reported the difficulties physiotherapy students experienced “interacting with other disciplines when working in a team” (Participant: Group D). This absence was evident by physiotherapy managers in the practice environment, where students experienced difficulties with the holistic management of the patient, as “holistic treatment requires exposure to all the disciplines that a patient would require as well as the integration of the different disciplines. A lot of physiotherapy graduates are going out to places and starting up departments without this knowledge” (Participant: Group F). While faculty was aware that “the role of the physiotherapist has changed, incorporating more of the psychosocial elements” (Faculty A), it was reported that the “curriculum does not address the whole person, but when we treat we encounter cultural, biological and social problems” (Faculty G) for which practitioners were underprepared.

## Discussion

Within an education context, Noddings (1986) states, “*I am first and foremost one -caring and, second, enactor of specialised functions. As teacher, I am, first, one-caring*”. Central to physiotherapy education and practice is the notion of caring. Interactions between student physiotherapists and patients on the one hand; and between students and physiotherapy practitioners variously teaching and supervising them on the other hand, offers a means to examine how caring is manifested (or not).

The construct of caring is expressed in different ways in student-faculty interactions. One interpretation for the relationship of care, which was constructed using the parent-child association, is the assumption that faculty display caring towards students in the same way that a parent would for their child, showing how apartheid constructions are still maintained. However, this relationship is underscored by a power differential, as the act of caring can be manipulated to exert authority as a maternal or paternal referent over the student. Through this relationship, boundaries are created that student physiotherapists, in turn, interpreted as the absence of caring by faculty. According to Noddings, many people do not understand the processes involved in bringing people into maturity and relationships of equality, with a lack of that knowledge resulting in them assuming that the process must be a dictatorial one (Belenky et al., 2000). Caring towards students was conflated with somewhat patronising parenting by physiotherapy faculty as was evidenced in the absence of listening, empathy and nurturing by students and managers in student-faculty interactions.

Another interpretation for authoritarian, hierarchical relations could be in response to the biomedical model and master-apprenticeship practitioner model that the physiotherapy curriculum structures (Wellard and Edwards, 1999). Physiotherapy theory and practise is supported by a particular view of ‘scientific’ knowledge that tends to exclude other legitimate forms of knowing, for example, social and emotional knowledge (Litchfield, 2002). This deficit was recognised by both students and faculty in the absence of a broader biopsychosocial approach to patient care, yet continued to be enacted in their relationships in the different spaces of the physiotherapy curriculum. Caring is expressed as physical competence and expertise within the biomedical model as it is based on scientific, measurable outcomes. Also, the position of authority of faculty is constructed at the student-faculty interface during knowledge and skills transfer, modelling a master-apprentice relationship. The competency-based curriculum tends to focus on mastery of skills in producing an expert practitioner. The healing nature of the profession constructs the image of the physiotherapist as a powerful being, an image that may have been modelled by faculty reinforcing unequal relations of power.

Both student and faculty described how boundaries were created that decreased the space for collaboration and dialogue on both personal and professional levels between them. Faculty not only dominated but did not forge relationships with students both within the formal official curriculum spaces as well as in the informal spaces, as noted by students in the lack of participation by academic staff at social events. This was further evidenced in the absence of opportunities to engage with other healthcare professionals outside of physiotherapy on an interdisciplinary platform. Both, however, also recognised how these hierarchical relations could be mitigated by engaging in dialogue and common tasks to build mutual respect and understanding and gave examples of how this could occur. Connection with faculty helps students feel like they belong at the institution (Duberstein, 2009).

Students and faculty socialised in racially segregated spaces, with few possibilities for creating racially diverse friendships. Little attention was given to “*promote participation in common social and intellectual activities between students from different backgrounds, and how to encourage and enable conversations between such students*” (Duncan, 2018). The need to create physical spaces to interact socially and informally, and have faculty participate in social activities has potential but remained under-explored. Space, according to Dixon and Janks (2018), is not neutral, it shapes a sense of self by acting as a space of belonging or a space of alienation, and affects the people who are encountered. The need to belong affects both university students and teachers, and positive teaching and learning environments, and interactions and relationships are likely to have beneficial impacts on participants and educational outcomes (Hagenauer and Volet, 2014).

A shift from one’s interest to those being cared for resonates with arguments that caring requires a capacity for empathy, and is demonstrated through a displacement of focus from self to others (Noddings, 1986; Collins, 2003). Cultivating empathy and practising perspective-taking can reduce bias and strengthen faculty-student relationships (Sparks, 2019). Listening, showing empathy and nurturing, were by and large not experienced by students, and several faculty conceded that these were lacking in their relationships with students. It was acknowledged that by listening, empathy may be engendered, but that the conditions for these were not produced in the curriculum.

Skills for inter-personal relationships are strongly influenced by positive role-models (Shapiro, 2008), which have been identified as a powerful teaching strategy that shapes the values, attitudes and behaviours of students (Harden & Crosby, 2000). Entrants to a new profession are greatly influenced by the examples presented before them, and internalise values of the profession by formal training and observing role-models. To create learning communities, teachers need to model behaviours and attitudes that are desired by students, such as being approachable, recognising talent and exhibiting good communication skills (Ernstzen, 2013). Practising acceptance, trust, openness and inclusion are central to caring relationships (O’Brien, 2010). Student physiotherapists are inducted into the profession through collegial relations and what is modelled by faculty. What can be observed in the findings is that these collegial interactions are influenced negatively by unequal relations of power that are depicted as authoritarian and hierarchical, which stymies the freedom to engage with the authority figure. These unequal relationships of knowledge and power failed to demonstrate mutual respect during exchanges between students and faculty.

In linking this analysis to the general higher education literature it may be argued that the themes discussed above are related to the affective and support dimensions as identified by Hagenauer and Volet (2014). The analysis also shows the dominance of the third dimension, that is, the extent to which these student-faculty relationships are context –dependent.

## Limitations

This study foregrounded the interactions of student, faculty and physiotherapy managers at one historically Black South African university which characterised the experiences of participants shaped by particular historical, geographical and demographic context relative to the location of the university. These experiences may be unlike those between physiotherapy or other health science faculty and students anywhere else in South Africa or globally and the findings cannot, therefore, be generalisable. Whilst this study has identified with other studies globally on generic elements of student-faculty interactions, it has expounded further on the contributing factors of the historical socio-political context on these interactions which may have little relevance to similar studies globally. The literature is limited on how caring is produced by faculty in health sciences curricula for modelling by students in the healthcare environment, particularly in educational contexts that display hierarchical relations. An important area for future research could be to compare how caring is produced during student-faculty interactions in health sciences curricula, particularly in contexts characterised by hierarchical relationships, and enacted in practice environments.

## Conclusions

The deeply hierarchical relations of power manifest between physiotherapy faculty and students, not only derived from elements of South Africa’s still present apartheid past but also a deeply entrenched positivist, biomedical model. The unsatisfactory nature of the relationships between faculty and students, according to some faculty, was influenced by the deficiencies of the physical environment and rested in the relationships amongst physiotherapy faculty themselves, the absence of cohesion and operating in isolation from each other, which was being transferred to students. Paternalistic relations were modelled in the physiotherapy curriculum, which in turn undermined the building of relationships with students as young adult learners and exacerbated the hierarchical student-academic relationship. Student-physiotherapists point to a divide or distance between themselves and faculty evidenced in the non-participation of the latter in student-related events, and in the instances of authoritarianism and disrespect that were displayed towards the student, including discriminatory practices.

There were considerable efforts in addressing dissonance or disjuncture in the relationships between the patients and students as an outcome of the curriculum. Caring relationships with patients may be theoretically espoused and practically enacted in the curriculum, but appear to be absent in the same physiotherapy curriculum spaces in the relationships between faculty and students. While the relationships are largely characterised negatively, a positive aspect is that students, faculty and managers are aware and have ideas to address the challenges they articulated. In a culturally diverse society, such as South Africa, maximising student’s opportunities to successfully navigate their tertiary education needs to include more than a good curriculum, and include a caring environment that nurtures the transition from student to caring professional. There is a need for more studies on such interventions in other health sciences curricula, and on how student-faculty interactions potentially influence student performance and their development as health professionals.

## Footnotes

1 Coloureds in South Africa are persons of mixed European (“White”) and African (“Black”) or Asian ancestry.

## References

[CR1] Belenky MF, Stanton AV, Mezirow J, Associates (2000). Inequality, development and connected knowing. Learning as transformation: Critical perspectives on a theory in progress.

[CR2] Benatar S (2015). The humanistic side of medical education. South African Medical Journal.

[CR3] Bergman R (2004). Caring for the ethical ideal: Nel Noddings on moral education. Journal of Moral Education.

[CR4] Bhana D (2014). Race matters and the emergence of class: Views from selected South African university students. South African Journal of Higher Education.

[CR5] Collins PH, Lincoln YS, Denzin NK (2003). Toward an afrocentric feminist epistemology. Turning points in qualitative research: Tying knots in a handkerchief.

[CR6] Cuseo J (2018). Student-faculty engagement. New Directions for Teaching and Learning.

[CR7] Demarrais, K., & Lapan, S. (2004). *Foundations for research: Methods of inquiry in education and the social sciences*.

[CR8] Department of Higher Education and Training. (2016). *Report on the second national higher education transformation summit international convention centre*. DurbanKwaZulu-Natal 15–17 October 2015.

[CR9] Dixon K, Janks H, Pattman R, Carolissen R (2018). Location and dislocation: Spatiality and transformation in higher education. Transforming transformation in research and teaching at South African Universities.

[CR10] Docan-Morgan T (2009). A typology of relational turning point events in college teacher-student relationships. Journal of the Scholarship of Teaching and Learning.

[CR11] Duberstein, A. (2009). Building student-faculty relationships. *Academic Advising Today, 32*(1). Retrieved from https://nacada.ksu.edu/.

[CR12] Duncan M, Pattman R, Carolissen R (2018). ‘Why did you choose to sit here?’ Interviews with people in same-race friendship groups at Stellenbosch University. Transforming transformation in research and teaching at South African Universities.

[CR13] Ernstzen DV (2013). Roles and attributes of physiotherapy clinical educators: Is there agreement between educators and students?. African Journal of Health Professions Education.

[CR14] Gore JM (1993). The Struggle for Pedagogies: Critical and feminist discourses as regimes of truth.

[CR15] Gough N, Mousley J, Gough N, Robson M, Colquhoun D (1998). Narrative and educational inquiry. Horizons, images and experiences: The research stories collection.

[CR16] Hagenauer G, Volet SE (2014). Teacher-student relationship at university: an important yet under-researched field. Oxford Review of Education.

[CR17] Harden RM, Crosby JR (2000). AMEE Education Guide No 20: The good teacher is more than a lecturer – The twelve roles of the teacher. Medical Teacher.

[CR33] Health Professions Council of South Africa. Core Competencies for Undergraduate Students in the Clinical Associate, Dentistry and Medical Teaching and Learning Programmes in South Africa. February 2014. http://www.hpcsa-blogs.co.za/

[CR34] Kallaway, P. (Ed.). (2002). *The history of education under apartheid: 1948–1994*. Peter Lang Inc. International Academic Publishers.

[CR18] Litchfield R, MacDougall C (2002). Professional issues for physiotherapists in family-centred and community-based settings. Australian Journal of Physiotherapy.

[CR19] Moyo M, Goodyear-Smith FA, Weller J, Robb G, Shulruf B (2016). Healthcare practitioners’ personal and professional values. Advances in Health Sciences Education.

[CR20] Noddings N (1986). Caring: A feminine approach to ethics and moral education.

[CR21] Noddings, N. (1989). Developing Models of Caring in the Professions. ERIC. ED 308594. Paper presented at the Annual Meeting of the American Educational Research Association (March 27–31, 1989).

[CR22] Noddings, N. (1991). Stories in dialogue: caring and interpersonal reasoning.

[CR23] Noddings N (2010). Moral education and caring. Theory and Research in Education.

[CR2000] Noddings N (2012). The caring relation in teaching. Oxford Review of Education.

[CR24] O’Brien LM (2010). Caring in the ivory tower. Teaching in Higher Education.

[CR25] Ramklass SS (2015). A framework for caring in physiotherapy education and practice. South African Family Practice.

[CR26] Reid S (2014). The ‘medical humanities’ in health sciences education in South Africa. South African Medical Journal.

[CR27] Ross, J. M. (2013). *The student-faculty relationship: An investigation of the interactions between students and faculty* (Doctoral dissertation, Gannon University).

[CR28] Shapiro J (2008). Walking a mile in their patients’ shoes: Empathy and othering in medical students’ education. Philosophy, Ethics, and Humanities in Medicine.

[CR29] Sparks SD (2019). Why teacher-student relationships matter: New findings shed light on best approaches. Education Week.

[CR30] Stone L, McLaren PL, Giarelli JM (1995). Feminist educational research and the issues of critical sufficiency. Critical theory and Educational Research.

[CR31] Verwey C, Quayle M (2012). Whiteness, racism, and Afrikaner identity in post-apartheid South Africa. African Affairs.

[CR32] Wellard R, Edwards H, Higgs J, Edwards H (1999). Curriculum Models for Educating Beginning Practitioners. Educating Beginning Practitioners: Challenges for Health Professional Education.

[CR35] Witherell, C., & Noddings, N. (Eds.). In & *Stories lives tell: Narrative and dialogue in education*. Teachers College Press

